# Comparative Efficacy of Selected Phytobiotics with Halquinol and Tetracycline on Gut Morphology, Ileal Digestibility, Cecal Microbiota Composition and Growth Performance in Broiler Chickens

**DOI:** 10.3390/ani10112150

**Published:** 2020-11-19

**Authors:** Muhammad Abdul Basit, Arifah Abdul Kadir, Teck Chwen Loh, Saleha Abdul Aziz, Annas Salleh, Zainul Amiruddin Zakaria, Sherifat Banke Idris

**Affiliations:** 1 Department of Preclinical Sciences, Faculty of Veterinary Medicine, Universiti Putra Malaysia, Serdang 43400, Selangor, Malaysia; bankidris67@gmail.com; 2 Department of Biosciences, Faculty of Veterinary Sciences, Bahauddin Zakariya University, Multan 60000, Punjab, Pakistan; 3 Department of Animal Sciences, Faculty of Agriculture, Universiti Putra Malaysia, Serdang 43400, Selangor, Malaysia; tcloh@upm.edu.my; 4 Department of Veterinary Pathology & Microbiology, Faculty of Veterinary Medicine, Universiti Putra Malaysia, Serdang 43400, Selangor, Malaysia; saleha@upm.edu.my; 5 Department of Veterinary Laboratory Diagnostics, Faculty of Veterinary Medicine, Universiti Putra Malaysia, Serdang 43400, Selangor, Malaysia; annas@upm.edu.my; 6 Department of Biomedical Science, Faculty of Medicine and Health Sciences, Universiti Putra Malaysia, Serdang 43400, Selangor, Malaysia; zaz@upm.edu.my; 7 Department of Veterinary Pharmacology and Toxicology, Faculty of Veterinary Medicine Usmanu Danfodiyo University, Skoto 2346, Nigeria

**Keywords:** antimicrobials, broiler chicken, cecal microbiota, gut health, halquinol, phytobiotics

## Abstract

**Simple Summary:**

Antimicrobial growth promoters (AGPs) are banned in Europe but still used in many countries including Asia. However, their indiscriminate use resulted in antibiotic-resistant bacterial strains that possibly transfer the resistant genes to the microorganisms pertinent to human health. Hence, it is essential to find alternatives that can improve the production performance in broiler chickens. In this scenario, phytobiotics or phytogenic feed additives (PFAs) are widely investigated to evaluate their influence on improving gut health, increasing digestibility, and thereby the growth performance. The present study is a continuity of our experiments on dietary inclusion of *Piper betle* and *Persicaria odorata* leaf meal and the first of its kind to evaluate the comparative efficacy of phytobiotics (*Piper betle* and *Persicaria odorata* leaf meal), with halquinol and tetracycline in broiler chickens. The current experiment findings indicated that, in comparison with the control group, either of the dietary treatments positively modulated the gut morphology, improved ileal digestibility, maintained the intestinal population of *Lactobacillus* and reduced the pathogenic bacteria such as *Staphylococcus aureus*, *Salmonella*, *Escherichia coli*, and *Clostridium* spp., thus improved the growth performance in broiler chickens.

**Abstract:**

The current experiment was designed to estimate the comparative efficacy of selected phytobiotics *Persicaria odorata* leaf meal (POLM) and *Piper betle* leaf meal (PBLM) with halquinol, and tetracycline in broiler chickens. The 150-day-old broiler chickens were randomly assigned to five dietary groups. The dietary supplementation groups were the basal diet (BD), which served as the negative control (NC), and BD + 0.2 g/kg tetracycline, which served as the positive control (PC); BD + 0.03 g/kg halquinol (HAL), BD + 8 g/kg POLM (Po8), and BD + 4 g/kg PBLM (Pb4) were the treatment groups. Growth performance, gut morphology, *ileal* digestibility, and cecal microbiota composition were measured. On day 21, the body weight gain (BWG) was enhanced (*p* < 0.05) in the broiler chickens fed on phytobiotics (Po8 and Pb4) relative to the NC group, however, on day 42 and in terms of overall growth performance, BWG was enhanced (*p* < 0.05 in diets (Po8, Pb4, HAL and PC) in comparison with the NC group. Conversely, feed conversion ratio (FCR) was recorded reduced (*p* < 0.05) in Pb4, Po8, HAL, and PC group in comparison with the NC group. Supplementation of phytobiotics (Po8 and Pb4), HAL and PC, positively improved the gut morphology compared to the NC group. Furthermore, the maximum (*p* < 0.05) villus height (VH) in duodenum and jejunum was observed in broilers fed on diet Pb4. Supplementation of phytobiotics, HAL and PC, improved (*p* < 0.05) the digestibility of dry matter (DM) (except for HAL), organic matter (OM), crude protein (CP), ether extract (EE), and ash compared to the NC group. Dietary supplementation of phytobiotics (Po8 and Pb4), HAL and PC, significantly reduced the *E. coli*, *Salmonella*, and *Staphylococcus aureus* (except for HAL) counts compared to the NC group. However, supplementation of Pb4 resulted in significantly decreased total anaerobic bacteria and *Clostridium* spp. counts compared to the NC group. In addition, supplementation of phytobiotics significantly increased the *Lactobacillus* count compared to HAL, PC, and NC groups. In conclusion, dietary supplementation of phytobiotics improved the gut morphology, positively modulated and maintained the dynamics of cecal microbiota with enhanced nutrient digestibility, thus, increased the growth performance. Based on current results, phytobiotics could be used as an alternative to AGPs for sustainable broiler chicken production.

## 1. Introduction

In several countries, except Europe, sub-therapeutic doses of antibiotics are added into diets to maintain gut health and increase the growth performance of broiler chickens [1,2]. However, consistent and indiscriminate use of antimicrobial growth promoters (AGPs) in broiler chickens’ diets resulted in the emergence of antimicrobial-resistant microorganisms, which may have the potential to disseminate the antibiotic-resistant pathogens among organisms [3,4]. Therefore, the European Union (EU) and many other countries have banned the use of AGPs in food-producing animals [5,6]. In this scenario, it is essential to find viable and potential alternatives for sustainable poultry production. Thus, extensive research is now focused on searching the alternative to AGPs with similar antimicrobial and growth-enhancing potential in broiler chickens [7,8].

At present, among several alternatives, phytobiotics or phytogenic feed additives (PFAs) have gained considerable attention [9]. The secondary bioactive compounds of phytobiotics possess comparable properties to synthetic AGPs [10] that can maintain the healthy gut and enhanced growth performance in broilers [11]. Numerous studies suggested that PFAs might be the potent and viable natural alternative to AGPs for poultry production [7,9,12,13]. Dietary supplementation of phytobiotics in broilers has considerable positive effects; their supplementation could improve gut health [14,15,16], positively modulate the dynamics of gut microbiota [17], and enhance feed efficiency [6,18], thus, increase growth performance [19,20,21,22,23]. Various studies have established the antibacterial efficacy of phytobiotics against pathogenic bacteria, such as *Clostridium* and *E. coli*, in broiler chickens [24,25].

The *Piper betle* is an essential herb of the Piperaceae family; it is primarily distributed in Asia and East Africa. In Malaysia and Indonesia, it is known as “*daun sirih*”. The *P. betle* has various properties, including anti-inflammatory [26], antioxidant [27], antimicrobial [28], and growth promoter [16]. Dried ground leaves of *P. betle* (100 g) contain 2.9 g of protein, 0.5 g fat, 2 g of crude fibre, 1.2 g ash, 5.6 g of carbohydrate, 9.8 mg of iron and 4.5 mg of vitamin C, [29]. This herb’s leaves have many traditional and medicinal uses [26]. The hydroxychavicol and eugenol are the main bioactive compounds present in *P. betle* leaf; these compounds have strong antibacterial properties [28,30].

Another essential herb is *Persicaria odorata* of the Polygonaceae family, and it is well distributed in Asia, including Malaysia, Indonesia, Singapore, and Brunei, known as “*daun kesum*” or “*daun laska*” [31]. This herb possesses various properties, including antimicrobial [32], antioxidant [33], and growth promoting [34]. The dried ground leaves of *P. odorata* contain 3.5% crude protein, 0.83% crude fat, 10.66% crude fibre, and 1.83% ash [35]. The myricetin and quercetin are essential bioactive compounds [36,37] and suggested to be responsible for antioxidant activity [38] and inhibit lipid peroxidation [39]. 

Halquinol is a quinolone but its mechanism of action is different from quinolones [40]. It is an antimicrobial, antifungal, and antiprotozoal substance [41,42]. Halquinol has a mechanism to slow peristalsis in the gut, thus helping to increase the absorption of nutrient from the gut [43]. Furthermore, it does induce a minimum bacterial resistance [42]. In countries such as Thailand, Brazil, India, Colombia, Indonesia, Bangladesh, and Peru it is used as a feed additive in poultry [41,44] and growth promoter in swine.

To the best of our knowledge, no previous literature was available about the comparative efficacy of POLM, PBLM with halquinol and tetracycline as an alternative to AGPs in broiler chickens. Limited studies were reported about supplementation of herbs containing tannins. As previously described, it was believed that tannins might have an anti-nutritional effect when supplemented in broiler chickens’ diet. However, multiple studies from the recent past revealed that the feed additives with appropriate low doses of tannins should enhance the growth performance in broiler chickens [16,45].

The current study was intended to evaluate the comparative efficacy of phytobiotics (POLM and PBLM) as an alternative feed additive to conventional AGPs in broiler chickens by estimating their effects on gut morphology, ileal digestibility, cecal microbiota composition, and growth performance measures.

## 2. Materials and Methods

### 2.1. Phytobiotics Source and Preparation

Fresh leaves of *Persicaria odorata* and *Piper betle* were harvested and processed for diets (POLM and PBLM) according to the previously described method [16]. Samples were obtained from the Herb Garden, Universiti Putra Malaysia (UPM). The collected fresh leaves were dried using an oven which was pre-set at 50 °C. These leaves were dried up to 72 h to get the homogeneous weight, then milled to get a fine powder and stored at 4 °C until further use.

### 2.2. Halquinol and Tetracycline 

The commercial product of halquinol (3 CARE FORTE) was supplied by M/S. Provimi Animal Nutrition India, while Tetracycline hydrochloride (cat:500105) was procured from Merck.

### 2.3. Experimental Design and Supplemented Diets

This study adhered to the guidelines approved by the Institutional Animal Care and Use Committee, UPM, in terms of animal care and experimental procedures with the reference number: (UPM/IACUC/AUP-R033/2018). One hundred and fifty 1-day-old male broilers (*Cobb*500) were obtained and wing-banned upon their arrival. The birds were randomly allocated into 5 treatment groups. Each experimental group contained five replicates of six birds each. The chickens were raised in cages with wire mesh floor (length 120 cm × width 120 cm × height 45 cm), which were placed in a conventional open-sided shed. The broiler chickens in all groups were reared under the same environmental and managemental conditions. The cyclic temperature in the house ranged between a maximum of 34 °C and a minimum of 24 °C, while the humidity ranged between a maximum of 91% and a minimum of 65% [34]. This study consisted of five dietary groups, and the diets were offered to the birds for 42 days (starter phase day 1–21 and finisher phase day 22–42). The basal diets Gold Coin^®^ (without any feed enzyme or premixing of antioxidant, antimicrobial, and anticoccidial drugs) were procured from feed supplier (Fajama Trading, No.52 JLN LP 1A/5 Taman Lestari Perdana 43300, Seri Kembangan, Selangor, Malaysia). The experimental diets were prepared, which met or exceeded NRC [46] recommendations (Table 1). The supplemented diets were the basal diet (BD), which served as the negative control (NC); BD + 0.2 g/kg tetracycline, which served as the positive control (PC); BD + 0.03 g/kg halquinol (HAL), BD + 8 g/kg POLM (Po8), and BD + 4 g/kg PBLM (Pb4), which were the treatment groups. The specific concentrations of diets were selected as earlier reported, PC [47], Po8 and Pb4 [16,34], while HAL as designated by [44]. There was free access to water and feed to the chicks during the entire experimental period, with continuous lighting. The vaccination and brooding of the birds were conducted according to [34].

### 2.4. Sample Collection

The chicks were weighed upon arrival to record the initial body weight at day 0. Then weekly, the feed intake (FI) and body weight (BW) were obtained during the entire experimental period. The body weight gain (BWG) and feed conversion ratio (FCR) were calculated to measure the growth performance of broilers. The mortality of broilers was noted when it occurred. At day 42, ten broiler chickens were randomly selected from each supplemented treatment (two from each replicate) and slaughtered according to the procedure described by “The Malaysian Standard (MS) 1500:2009” [48]. From each slaughtered bird, the intestinal samples were obtained to measure nutrient digestibility, gut morphology, and cecal microbiota composition, as well as *ileal* digesta, which was collected from each bird to measure the apparent ileal digestibility. 

### 2.5. Nutrient Digestibility

To measure nutrient digestibility, titanium dioxide (TiO_2_), an indigestible maker, was added in all experimental treatment groups at a rate of 3 g/kg of feed at days 39–42 [49]. The ileal digesta samples were obtained from the slaughtered birds and then stored at −80 °C until analysis. The ‘‘TiO_2_” concentrations of digesta and feed were measured [50]. Meanwhile, the crude protein (CP), dry matter (DM), organic matter (OM), ether extract (EE), and ash of digesta and feed were determined using proximate analysis procedure AOAC [51]. The apparent ileal digestibility (AID) of DM, OM, CP, EE and Ash was determined using TiO_2_ ratios in the feed and ileal digesta according to [52] using Formula (1):

Apparent ileal digestibility (AID) of nutrient = 100 – ((% TiO_2_ in feed/%TiO_2_ in ileal content) × (% of nutrient in ileal content/%nutrient in feed × 100)). (1)

### 2.6. Histomorphology of Intestine 

Histomorphometry of gut was performed according to the procedure as designated by Alshelmani et al. [49]. Approximately 5 cm intestinal portions of duodenum, ileum, and jejunum were obtained, followed by flushing with buffered saline before to be fixed in formalin solution (10%). The intestinal segments were cut approximately into 3.5 mm sections. These tissue sections of the intestine were then subjected to a series of dehydration sequences using tissue processor with automation (ASP300, Leica biosystem, Wetzlar, Germany). The embedding of gut sections was performed using a paraffin embedding station (EG1150 II, Leica, Wetzlar, Germany). The intestinal sections were trimmed up to 4–5 μm with a sectioning rotary microtome (RM2045, Leica Wetzlar, Germany). The staining of tissue sections was performed using haematoxylin and eosin staining procedures. The gut morphometric parameters were measured using the light microscope mounted with a digital camera (Leica DM LB2, Wetzlar, Germany). The 12 villi and crypts per each tissue were examined for analysis and observations. A method designated by Touchette et al. [53] was used to measure the villus height (VH) and crypt depth (CD) using the Image-Pro Plus software. 

### 2.7. Cecal Microbiota Composition and Cecal pH

The cecal contents were collected aseptically into the sterilised plastic bags from the ceca of slaughtered birds. The cecal contents were immediately stored at −20 °C and processed within 24 h for the cecal microbiota population count. The collected cecal digesta homogenates were serially diluted from 10^−1^ to 10^−7^. Selected agar media were used for the enumeration of targeted bacterial groups. The *Escherichia. coli* was enumerated using Brilliance E.coli/coliform media (CM 0956; Oxoid, UK), total anaerobic was cultured using Wilkins Chalgren Agar (CM 0619; Oxoid, UK), *Pseudomonas aeruginosa* was cultured using Pseudomonas Cetrimide agar (CM 0579; Oxoid, UK) *Salmonella* was cultured using XLD agar (CM 0469; Oxoid, UK), *Clostridium* was cultured using reinforced clostridial agar (CM0149; Oxoid, UK), *Staphylococcus aureus* was cultured using Baird parker agar (CM 0275; Oxoid, UK) and *Lactobacillus* was cultured using MRS agar (CM0361; Oxoid, UK). The culture plates were then incubated at 37 °C for a period of 48 h. In the case of clostridia plates, incubation was performed anaerobically at 37 °C for a period of 48 h. A colony counter was used for the enumeration of visible colonies, and the results were expressed as log_10_ CFU/g of cecal digesta. The *cecum* pH was measured using a digital pH meter (AG8603, Benchtop pH Meter; Mettler Toledo, Switzerland).

### 2.8. Statistical Analysis 

The obtained data were analysed using Statistical Analysis System software (SAS) version 9.4 [54] (SAS Institute Inc., Cary, NC, USA) using one-way ANOVA. Group differences were then elucidated and compared by Duncan’s multiple range test. Statistically significant differences were considered at *p* < 0.05.

## 3. Results

### 3.1. Growth Performance

On day 21, broiler chickens raised on diets Po8 and Pb4 showed enhanced (*p* < 0.05) body weight gain (BWG) relative to the broilers raised on NC diet. On day 42, dietary supplementation of Po8, Pb4, HAL, and PC increased (*p* < 0.05) BWG relative to NC group. Considering the overall growth performance, BWG was higher (*p* < 0.05) in Po8, Pb4, HAL, and PC, compared to the NC group; however, maximum improved (*p* < 0.05) BWG was recorded in Pb4 group. Conversely, decreased (*p* < 0.05) feed conversion ratio (FCR) was noted throughout the experimental period in supplemented groups Po8, Pb4, Hal, and PC compared to the NC group. Regarding feed intake (FI), no significant differences were observed among experimental broiler chickens in the entire experimental period (Table 2).

### 3.2. Nutrient Digestibility

Dietary inclusion of Po8, Pb4, HAL, and PC, improved (*p* < 0.05) the apparent ileal digestibility of crude protein (CP), dry matter (DM) (except for HAL), organic matter (OM), ether extract (EE), and ash in comparison with the NC group (Table 3).

### 3.3. Histomorphology of Intestine 

Supplementation of phytobiotics (Po8 and Pb4), HAL, and PC resulted in improved (*p* < 0.05) villus height (VH) in the jejunum and duodenum compared with NC; regarding ileum increased VH was noted in phytobiotics supplemented group and PC group relative to HAL and NC group. Furthermore, Pb4 supplementation resulted in maximumly improved (*p* < 0.05) VH in the jejunum and duodenum. Conversely, broilers fed on supplemented diets Po8, Pb4, HAL, and PC significantly reduced (*p* < 0.05) the crypt depth (CD) in the duodenum and ileum relative to the NC group; however, no difference (*p* > 0.05) was noted for CD in the jejunum among the experimental broiler chickens. Significantly increased villus height to crypt depth ratio (VH: CD) ratio was recorded in the duodenum, *ileum*, and jejunum of broilers fed on, phytobiotics, HAL, and PC in comparison with the broiler chickens fed on the NC diet (Table 4). 

### 3.4. Cecal Microbiota 

The composition of the cecal microbial population of broiler chickens fed on phytobiotics, HAL, tetracycline (PC), and basal diet (NC) is shown in Table 5. Dietary supplementation of phytobiotics (Po8 and Pb4), HAL, and PC decreased (*p* < 0.0001) the *Escherichia coli* (*E. coli*) count relative to the NC group. Supplementation of Pb4 decreased (*p* < 0.05) the total anaerobic bacteria compared to HAL and the NC groups. Furthermore, except for HAL, supplementation of phytobiotics and PC significantly decreased the *Staphylococcus aureus* count relative to the NC group; however, the lowest (*p* < 0.0001) *Staphylococcus aureus* count was noted in PC and Pb4 groups compared to Po8 group. In addition, no significant difference was observed in the *Pseudomonas aeruginosa* count between phytobiotic, HAL, PC, and NC groups. The *Clostridium* counts were decreased (*p* < 0.003) with supplementation of Pb4 and PC compared to Po8, HAL, and NC groups. Dietary supplementation (phytobiotic, HAL, PC, and NC) decreased (*p* < 0.0001) the *Salmonella* count in comparison with the NC group. The significantly lowest (*p* < 0.0001) *Salmonella* counts were recorded in Pb4 and PC group compared to Po8, HAL, and NC groups. However, supplementation of Po8 and HAL resulted in a significantly decreased *Salmonella* count in comparison with the NC group. Furthermore, supplementation of phytobiotics increased (*p* < 0.0001) the *Lactobacillus* count compared to HAL, PC, and NC groups; however, two latter groups, HAL and PC, were not significantly different compared to the NC group. The cecum pH was significantly decreased (*p* < 0.001) in supplemented groups Po8, Pb4, HAL and PC, in comparison with the NC group.

## 4. Discussion

### 4.1. Growth Performance

Phytobiotics, such as natural herbs and their secondary bioactive compound, possess the considerable potential to enhance the growth performance of broilers by increasing their feed efficiency [10,30,55]. Phytobiotics possess antioxidant, antimicrobial, and anti-inflammatory properties. Their use as a feed additive can positively enhance the intestinal health, increase the absorption and digestibility of nutrients; hence, improve feed efficiency [18,56]. 

In this study on day 21, the improved (*p* < 0.05) BWG was noted in phytobiotic (Po8 and Pb4) groups in comparison with the control group. Furthermore, on day 42, increased BWG was observed in Po8, Pb4, HAL, and PC groups relative to the NC group. Conversely, FCR was decreased (*p* < 0.05) in Po8, Pb4, HAL, and PC group in comparison with the NC group. Hassan et al. [57] endorsed current study results; he reported that the supplementation of the phytobiotic (Sanguinarine) enhanced the BWG and significantly decreased the FCR parallel to conventional AGPs. Various other studies showed that in-feed supplementation of phytobiotics or PFAs in broiler chickens improved the BWG and decreased FCR, Furthermore, these studies indicated that the phytobiotics might be used as an alternative to AGPs [12,55].

Herbs possess flavonoids, tannins, polyphenols, and alkaloids; these secondary metabolites have the potential to enhance the health of birds [56]. The current study results might be due to the presence of secondary metabolites, mainly due to the presence of bioactive compounds like quercetin and eugenol. Both bioactive compounds have been successfully quantified—quercetin from POLM, while the eugenol from PBLM (unpublished data). The polyphenol, such as flavonoid (quercetin), and phenolic compounds such as eugenol, are presumed to increase the growth measures in broilers when supplemented as feed additives [20,58]. 

The eugenol can increase appetite [59] and stimulate digestive enzymes secretions [60]. In the current study, the broiler chickens fed on diet Pb4, which contains tannins and secondary bioactive compound eugenol, showed enhanced growth performance. These results are in line with Paraskeuas et al. [20] who stated that the dietary inclusion of PFAs (eugenol, menthol, and anethol) in broiler birds increase the growth measures. Likewise, Oso et al. [45] described that dietary inclusion of PFAs in the form of a mixture including *Piper betle*, *Piper nigrum*, and *Cynodon dactylon* improved weight gain and decreased FCR. Phytobiotics containing tannins are previously considered not suitable for monogastric animals, including poultry [61]. The feed palatability of birds might be decreased when supplemented with higher concentrations of tannins [62]; however, tannins positively influence the growth performance of birds when supplemented in appropriate low doses [16,63]. Balanced supplementation of tannins enhanced the digestion of nutrients, which can positively modulate the intestinal microbiota, which helps to increase the production performance of broiler chickens [61,64].

Flavonoids such as quercetin (bioactive compound of POLM), which is a flavone, enhance the secretion of insulin-like growth factor-1, which results due to the increased concentration of growth hormone and stimulation of hepatic growth hormone receptor [65]. Thus, the supplementation of quercetin improved production performance in broilers [66]. In the present study, broilers fed on the diet Po8 showed increased growth performance compared to the NC group; these outcomes are parallel to Aroche et al. [67] and Zhou et al. [68] where they reported that supplementation of PFAs mix powder and *baicalein* flavonoids resulted in increased BWG and improved FCR in broiler chickens. 

The present study results showed that in terms of overall growth performance, BWG was significantly enhanced (*p* < 0.05) in Po8, Pb4, HAL, and PC supplemented groups relative to NC group; however, maximumly increased (*p* < 0.05) BWG was observed in birds raised on dietary supplementation Pb4. On the other hand, FCR was significantly decreased in Po8, Pb4, HAL, and PC groups compared to the NC group. Current study results are supported by various other studies supplementing phytobiotics to broiler chickens [20,23].

Conclusively, dietary inclusion of phytobiotics (PBLM and POLM) in broilers showed improved growth performance. In addition, this enhanced growth performance in broiler chickens was parallel to the PC (AGPs). Numerous previous studies showed similar outcomes where phytobiotics were supplemented to broilers and showed parallel results to AGPs [7,21,30,57,69]. Based on current study findings, it was concluded that phytobiotics (PBLM and POLM) could be used as alternative growth promoters to AGPs in broiler chickens. 

### 4.2. Nutrient Digestibility

Phytobiotics, such as herbs, can stimulate the secretion of enzymes [70] and positively modulate the intestine by increasing the VH and their surface area [45]. Thus, enhance the nutrient digestion and absorption from the gut of broiler chickens [1,19]. In the present experiment, the broiler chickens fed on diets Po8, Pb4, HAL and AGPs showed improved (*p* < 0.05) digestibility of crude protein (CP), dry matter (DM) (except for HAL), organic matter (OM), ether extract (EE), and ash compared to the NC group. The current study results added information to previous studies that showed a positive influence of phytobiotics to increase absorption and digestibility of nutrients in broiler chickens [16,18,71].

Phytobiotics can enhance palatability, modify the intestinal walls, and improve nutrient absorption. In addition, it can reduce the pathogenic microbial load and inflammation along with the increased secretion of digestion enzymes, thus, it can enhance the nutrient digestibility [1,17]. The present study results showed that supplementation of phytobiotics enhanced the apparent ileal digestibility of DM, OM, EE, CP, and ash. These results are similar to the outcome of Hassan et al. [72] and Farahat et al. [73], where supplementation of phytobiotics, artichoke (*Cynara scolymus*), and grape seed tannin in broiler diets improved digestibility of DM, EE, and CP. Halquinol can slow peristalsis in the gut [43]; this slowed passage of food in the gut may help in enhanced absorption.

In conclusion, the supplementation of Po8 and Pb4 increased the digestibility of nutrients. The bioactive compounds, such as flavonoids and polyphenols, present in phytobiotics (Pb4 and Po8) positively modulate the gut and improve nutrient digestibility, thus, enhancing the growth performance of broiler chickens [9,74].

### 4.3. Gut Morphology

The gut is an important organ that contributes to some important functions, including digestion and host defence. Any impairment in gut function can alter the digestibility of nutrients; thus, health and growth performance of chickens might be declined [75]. The VH and CD are significant factors of the gut that indicate its function, as well as the health status of animals [76]. Increased VH and decreased CD with bigger surface area, can help to increase the absorption of nutrients. Furthermore, increased VH enhances the transport of nutrients, thus, improves the growth performance of birds [77]. Broiler chickens are fast-growing, their intestinal epithelia have an ability to increase nutrient absorption and then conversion of absorbed nutrients into body mass [75].

The current study showed that supplementation of phytobiotics (Po8 and Pb4), HAL, and PC diets resulted in increased (*p* < 0.05) VH in the duodenum as well as in the jejunum relative to the NC group. Furthermore, Pb4 supplementation resulted in maximum (*p* < 0.05) VH in the duodenum and jejunum of broiler chickens. Conversely, broilers fed on supplemented diets Po8, Pb4, HAL, and PC exhibited decreased (*p* < 0.05) CD in the duodenum and ileum compared to the NC group. On the other hand, significantly higher VH: CD ratios were observed in the duodenum, jejunum and ileum of broilers fed on Po8, Pb4, HAL, and PC diets in comparison with the broiler chickens fed on the NC diet. The current outcomes are in line with the idea that in-feed supplementation of phytobiotics in broiler chickens can positively improve the gut function by stimulating the secretion of gut mucus, changing the morphological architecture of intestine, such as increase in VH and decrease in CD [74]. Thus, in-feed inclusion of phytobiotics in broiler chickens enhanced the intestinal function and maintained the healthy gut [17,56]. Murugesan et al. [9] found that phytobiotics can increase VH, reduce inflammatory events, and enhance the absorption efficiency in broiler chickens. Soliman et al. [71] and Oso et al. [45] endorsed current study findings where supplementation of PFAs had significantly increase VH and decrease CD. As with the present study results, the positive influence of phytobiotics on gut morphology in broiler chickens was reported in several studies [14,17,24]. Various other studies endorsed the present study findings, where dietary supplementation of phytobiotics possessing polyphenols, including flavonoids and phenolic compounds, modulated the gut health through improved gut microarchitecture [25,56].

The intestinal crypts are considered as a source of epithelial cells in the gut villi; additionally, the CD is having a direct correlation with the epithelial cell turnover. The deeper crypts reflect increased cellular turn over and needed more energy to uphold the gut function in the broiler birds [78]. The present study results show that supplementation of phytobiotics leads to shallow crypts, which designate healthy intestine with reduced cellular turnover. A cellular turnover is an energy-utilising event; shallow crypts resulted in decreased cellular turnover, thus reduced energy utilisation which might be used in growth events of broiler chickens. These findings are endorsed by earlier reports that showed increased VH, and shallow crypts indicated enhanced growth performance of birds [14,25,71].

Conclusively supplementation of phytobiotics (Pb4 and Po8) positively modulate the gut, the increased VH, and decreased CD, recorded in dietary groups, might be an explanation for improved growth performance in broiler chickens.

### 4.4. Cecal Microbiota

Gut microbiota has a significant effect on the nutritional status, metabolism, and health of the broilers [79]. The mechanism by which AGPs improve growth performance in birds is still unclear; however, it is believed that AGPs enhanced the growth performance of birds through modulation of gut microbiota [80]. A healthy gut with a balanced microbiota population is important to enhance feed efficiency, protection against pathogens, and to improve growth performance of birds [81]. Beneficial microbes such as *Lactobacillus* are important for gut functions, including metabolism, energy transfer, and growth [82]. On the other hand, an increased population of harmful pathogens such as *E. coli*, *Salmonella*, and *Clostridium* can change the balanced ecosystem of the gut and result in compromised growth [83].

Phytobiotics can reduce pathogenic microbial growth [84], can favourably improve microbiota balance and improve intestinal function [17]. Thus, phytobiotics can enhance the health status and performance of the birds [85]. Several studies reported the antimicrobial activity of PFAs possessing polyphenols, including flavonoid (quercetin) and eugenol [14,59]. Antimicrobial activity is one of the properties of phytobiotics when supplemented as growth promoters in the ration of broilers [86].

In the current study, the dietary supplementation of phytobiotics (Po8 and Pb4), HAL, and PC decreased (*p* < 0.0001) the *E. coli* count relative to the NC group. Furthermore, the supplementation of phytobiotics and PC significantly decreased the *Staphylococcus aureus* count in comparison with the NC group. Dietary supplementation of Pb4 reduced (*p* < 0.05) the total anaerobic bacteria compared to HAL and NC groups. The *Clostridium* counts were reduced (*p* < 0.003) with dietary inclusion of Pb4 and PC compared to Po8, HAL, and NC group. In addition, dietary supplementation of Po8 and Pb4, HAL, and PC reduced (*p* < 0.0001) the *Salmonella* count compared to the NC group; however, the significantly lowest (*p* < 0.0001) *Salmonella* counts were recorded in Pb4 and PC groups. Furthermore, supplementation of Po8 and Pb4 increased (*p* < 0.0001) the *Lactobacillus* count compared to HAL, PC, and NC groups.

The present study results are in good agreement with the work of Wati et al. [12] who reported that the supplementation of PFAs and bacitracin methylene disalicylate in broiler chickens’ diet were equally effective. In addition, the PFAs significantly decreased the *Salmonella*, *E. coli* and *Clostridium* counts compared to the NC; conversely, significantly increased *Lactobacillus* count was recorded in the PFA group in comparison with PC and NC groups. Very much similar results were described by Murugesan et al. [9] where dietary supplementation of bacitracin and phytobiotics in broilers significantly reduced the total anaerobic bacteria and *Clostridium* counts, however, only phytobiotics reduced the *Coliforms* count. Furthermore, phytobiotics significantly increased the *Lactobacillus* count compared to PC and NC groups. Several studies endorsed the current study findings of supplemented groups Po8 and Pb4, where supplementation of the phytobiotics leaf meal in broilers’ diet possessing secondary metabolites polyphenols such as flavonoid and phenolic compound can positively modulate the microbiota balance [22,87,88].

To understand the detailed effects of AGPs and phytobiotics on chicken intestinal microbiota, it is necessary to know the mechanisms of growth promotion [89]. In the current study, the phytobiotics (especially Pb4) showed better results than the HAL, PC and NC groups. In addition, supplementation of Pb4 was superior in the modulation of gut microbiota compared to all other treatments. Previous studies showed that the presence of tannins in *P. betle* [28], and their balanced dose can improve the gut microbiota and positively modulate the gut, hence, enhance the growth performance in broilers [13,16,61,63,90]. The antimicrobial mechanism of action of tannins is proposed to limit the metal ion and interact with the cell membrane in terms of complexes. Furthermore, tannins can morphologically change the cell wall and also increase the permeability of cell membrane [64,91]. The number of pathogenic microorganisms, including *E. coli*, *Salmonella*, and *staphylococcus*, was reported sensitive to tannins [64,92,93]. The tannin mechanism of iron chelation resulted in less metal ion availability, which may inhibit microbial growth other than *Lactobacillus*, as these microorganisms do not need iron for their growth [94].

The secondary bioactive compounds quercetin and eugenol were successfully quantified from POLM and PBLM, respectively (unpublished data). Previous studies revealed that eugenol and quercetin possess strong antimicrobial properties [95,96,97,98]. Eugenol has shown antimicrobial activity against Gram-negative bacteria, including *Salmonella*, *E. coli*, and *P. aeruginosa,* also, against Gram-positive microorganisms, including *Staphylococcus aureus* and *Clostridium* [97,99]. The proposed antimicrobial mechanism of eugenol is increasing the permeability of the cell membrane and has disruptive action on the cytoplasmic membrane of bacteria [100]. Furthermore, the dietary supplementation of eugenol in broiler chickens resulted in the reduced pathogenic microbial count of bacteria like *Salmonella* [101,102]. On the other hand, Quercetin has exhibited bacteriostatic activity against different strains of bacteria, such as *S. aureus* and *E. coli* [95,103]. The current study results are parallel to Wang et al. [96], where in-feed supplementation of quercetin in broiler chickens resulted in a significantly reduced count in the microbial population of *S. aureus*, *E. coli* and *Salmonella* and significantly increased *Lactobacillus* count compared to the control group. These results are endorsed by other studies that showed that quercetin and other flavonoids had an inhibitory effect on *E. coli* [64], *S. aureus* [104], and *Salmonella* [105]. Additionally, quercetin and other flavonoids play an important role in increasing *Lactobacillus* count [106]. The proposed antimicrobial mechanism of flavonoids, such as quercetin, showed increased permeability of cell walls and cell membranes [107].

In the current study, the supplementation of phytobiotics (Po8 and Pb4), HAL, and PC significantly reduced the cecal pH; however, the least (*p* < 0.05) pH values were recorded in the cecum of broiler chickens fed on phytobiotics supplemented diets. These results might be due to an increased *Lactobacillus* count in phytobiotics treated groups, which helps to decrease gut pH and modulate an environment that is not favourable for the growth of pathogenic microorganisms. The current idea is endorsed by Ripon et al. [108], where supplementation of PFAs lowering the gut pH, thus inhibiting the pathogenic bacteria as positive modulation of gut microbiota, has a primary role in host health. A similar idea was reported by Saki et al. [109].

In conclusion, supplementation of phytobiotics positively modulated the gut health by increasing the beneficial microbes, such as *Lactobacillus*, and restricting the growth of pathogenic bacteria, including *E. coli*, *Staphylococcus aureus*, *Salmonella*, and *Clostridium*. Current results are supported by a variety of other studies that reported that phytobiotics supplementation in broiler chickens significantly reduced the pathogenic bacteria including *E. coli*, *Salmonella* and *Clostridium* and significantly increased the beneficial bacteria (*Lactobacillus)* [9,14,24,71].

## 5. Conclusions

In conclusion, dietary supplementation of phytobiotics (Po8 and Pb4) positively improved the gut microarchitecture, modulated the dynamics of cecal microbiota with enhanced nutrient digestibility, thus, improved the growth performance. In comparison with NC, HAL and PC phytobiotics showed improved growth performance, increased intestinal villi height, reduced pathogenic bacteria count (*E. coli*, *Staphylococcus aureus*, *Salmonella*, and *Clostridium*) and enhanced the beneficial microbes such as *Lactobacillus.* Moreover, among the phytobiotics, Pb4 showed superior results. Based on the present findings, phytobiotics, especially Pb4, could be used as an alternative to conventional AGPs for sustainable broiler chicken production.

## Figures and Tables

**Table 1:** Ingredient (% as feed) and nutritional analysis feed used in the current study.

Ingredients %	Starter					Finisher				
	NC	PC	HAL	Po8	Pb4	NC	PC	HAL	Po8	Pb4
Corn	54.40	54.40	54.40	54.40	54.40	58.95	58.95	58.95	58.95	58.95
Soybean Meal (SBM) (44%)	33.90	33.90	33.90	33.90	33.90	28.00	28.00	28.00	28.00	28.00
Palm Oil	2.64	2.64	2.64	2.64	2.64	4.14	4.14	4.14	4.14	4.14
Dicalcium Phosphate	1.09	1.09	1.09	1.09	1.09	0.84	0.84	0.84	0.84	0.84
Choline Chloride	0.11	0.11	0.11	0.11	0.11	0.10	0.10	0.10	0.10	0.10
Salt	0.38	0.38	0.38	0.38	0.38	0.28	0.28	0.28	0.28	0.28
DL-Methionine	0.18	0.18	0.18	0.18	0.18	0.18	0.18	0.18	0.18	0.18
L-Lysine	0.34	0.34	0.34	0.34	0.34	0.34	0.34	0.34	0.34	0.34
Fish Meal	5.33	5.33	5.33	5.33	5.33	5.74	5.74	5.74	5.74	5.74
Limestone	1.06	1.06	1.06	1.06	1.06	0.86	0.86	0.86	0.86	0.86
Mineral Mix ^1^	0.28	0.28	0.28	0.28	0.28	0.28	0.28	0.28	0.28	0.28
Vitamin Mix ^2^	0.29	0.29	0.29	0.29	0.29	0.29	0.29	0.29	0.29	0.29
**Treatments g/kg of Feed ^3^**										
Tetracycline (PC)	0	0.2	0	0	0	0	0.2	0	0	0
Halquinol (HAL)	0	0	0.03	0	0	0	0	0.03	0	0
POLM (Po8)	0	0	0	8	0	0	0	0	8	0
PBLM (Pb4)	0	0	0	0	4	0	0	0	0	4
**Calculated Analysis (%) ^4^**										
Metabolisable Energy (ME) MJ/kg	13.10	13.10	13.10	13.10	13.10	13.40	13.40	13.40	13.40	13.40
Crude Protein, %	22.01	22.20	22.09	22.4	21.981	20.10	20.09	19.93	19.95	20.05
Ether Extract, %	5.27	5.22	5.29	5.22	5.18	7.01	7.10	7.21	7.09	7.12
Calcium, %	0.99	0.99	0.99	0.99	0.99	0.90	0.90	0.90	0.90	0.90
Available P, %	0.42	0.42	0.42	0.42	0.42	0.36	0.36	0.36	0.36	0.36

^1^ Premix administered minerals per kg of dietary feed: Zinc, 100.01 (mg); Iron, 120.0 (mg); I, 0.8 (mg); Mg, 16.0 (mg); Cu, 19.99 (mg); Co, 0.6 (mg).^2^ Premix administered vitamins per (kg) of dietary feed: Vitamin E, 0.02 (mg); Vitamin A (retinol), 1950 (μg); Vitamin K (menadione) 1.33 (mg); Vitamin D_3_ 30 (μg); Vitamin B_3_ 24 (mg); Biotin, 0.03 (mg); Riboflavin, 2.0 (mg); Vitamin B_12_, 0.03 (mg); Vitamin B_1_, 0.83 (mg); Vitamin B_6_, 1.37 (mg); Calcium d-Panthothenate, 3.69 (mg); Folic acid, 0.33 (mg). ^3^ NC: (basal diet; negative control); PC: (basal diet + 0.2 g/kg tetracycline; positive control); HAL: (basal diet + 0.03 g/kg halquinol); Po8: (basal diet + POLM 8 g/kg); Pb4 (basal diet + PBLM 4 g/kg). ^4^ Calculated according to NRC [46].

**Table 2:** Growth performance and mortality of broilers supplemented with phytobiotics, HAL and antimicrobial growth promoters (AGPs).

Parameters	Treatments						
	NC	PC	HAL	Po8	Pb4	SEM	*p*-Value
**1–21 days**							
Body weight gain (g)	631.2 ^b^	667.3 ^ab^	666.8 ^ab^	675.3 ^a^	688.7 ^a^	6.41	0.04
Feed Intake (g/bird)	1030.60	1023.7	1021.6	1029.0	1028.5	6.19	0.98
FCR	1.63 ^a^	1.53 ^b^	1.53 ^b^	1.52 ^b^	1.50 ^b^	0.02	0.03
**21–42 days**							
Body weight gain (g)	1658.5 ^b^	1716.3 ^a^	1714.6 ^a^	1722.1 ^a^	1748.4 ^a^	9.47	0.03
Feed Intake (g/bird)	3010.0	3011.2	3013.0	3014.0	3016.0	8.36	0.99
FCR	1.82 ^a^	1.75 ^b^	1.76 ^b^	1.75 ^b^	1.73 ^b^	0.01	0.02
**1–42 days**							
Body weight gain (g)	2291.7 ^c^	2383.6 ^b^	2381.4 ^b^	2397.4 ^b^	2437.5 ^a^	11.09	<0.0001
Feed Intake (g/bird)	4043.0	4035.0	4036.2	4043.0	4045.0	11.01	0.99
FCR	1.76 ^a^	1.69 ^b^	1.69 ^b^	1.69 ^b^	1.66 ^b^	0.01	<0.0001
Mortality rate (%) day 1–42							
	10	3.66	6.66	3.66	0.00	-	-

Note: ^a–c^ means with different superscripts in the same row differ significantly(*p* < 0.05). NC: (basal diet; negative control); PC: (BD + 0.2 g/kg tetracycline; positive control); HAL: (BD + 0.03 g/kg halquinol); Po8: (BD + POLM 8 g/kg); Pb4 (BD + PBLM 4 g/kg). FCR, feed conversion ratio. SEM: standard error of mean.

**Table 3:** Nutrient digestibility of broilers supplemented with phytobiotics, HAL and AGPs.

Treatment							
Parameter	NC	PC	HAL	Po8	Pb4	SEM	*p*-Value
Dry matter (DM)	70.00 ^b^	76.92 ^a^	75.63 ^ab^	77.31 ^a^	78.68 ^a^	0.96	0.03
Organic matter (OM)	71.50 ^b^	78.20 ^a^	77.05 ^a^	78.34 ^a^	79.10 ^a^	0.93	0.01
Ether extract (EE)	67.76 ^b^	74.94 ^a^	74.25 ^a^	75.17 ^a^	75.3 ^a^	0.93	0.04
Crude protein (CP)	69.87 ^b^	76.26 ^a^	76.05 ^a^	77.72 ^a^	77.19 ^a^	0.92	0.04
Ash	32.20 ^b^	35.32 ^a^	35.03 ^a^	35.08 ^a^	35.47 ^a^	0.39	0.03

Note: ^a,b^ means with different superscripts in the same row indicate significant difference, (*p* < 0.05). NC: (basal diet; negative control); PC: (BD + 0.2 g/kg tetracycline; positive control); HAL: (BD + 0.03 g/kg halquinol); Po8: (BD + POLM 8 g/kg); Pb4 (BD + PBLM 4 g/kg). SEM: standard error of mean.

**Table 4:** Gut morphology of broilers supplemented with phytobiotics, HAL and AGPs.

Treatment							
Parameter	NC	PC	HAL	Po8	Pb4	SEM	*p*-Value
**Villus height**							
Duodenum	1383.8 ^c^	1460.1 ^b^	1451.7 ^b^	1476.4 ^ab^	1489.0 ^a^	6.30	<0.0001
Jejunum	970.8 ^c^	1040.2 ^b^	1030.1 ^b^	1043.6 ^ab^	1063.0 ^a^	5.43	<0.0001
Ileum	642.8 ^b^	663.8 ^a^	655.8 ^ab^	664.7 ^a^	667.4 ^a^	2.85	0.04
**Crypt depth**							
Duodenum	185.5 ^a^	168.5 ^b^	167.6 ^b^	171.5 ^b^	169.9 ^b^	2.01	0.02
Jejunum	143.6	138.9	138.7	138.3	137.8	1.52	0.76
Ileum	135.0 ^a^	124.2 ^b^	123.1 ^b^	123.9 ^b^	123.2 ^b^	1.47	0.03
**VH: CD**							
Duodenum	7.5 ^b^	8.7 ^a^	8.7 ^a^	8.7 ^a^	8.8 ^a^	0.13	0.002
Jejunum	6.8 ^b^	7.5 ^a^	7.5 ^ab^	7.6 ^a^	7.8 ^a^	0.12	0.04
Ileum	4.80 ^b^	5.4 ^a^	5.4 ^a^	5.4 ^a^	5.5 ^a^	0.07	0.04

Note: ^a–c^ means with different superscripts in the same row indicate significant difference, (*p* < 0.05). NC: (basal diet; negative control); PC: (BD + 0.2 g/kg tetracycline; positive control); HAL: (BD + 0.03 g/kg halquinol); Po8: (BD + POLM 8 g/kg); Pb4 (BD + PBLM 4 g/kg). SEM: standard error of mean.

**Table 5:** Cecal microbiota composition of broilers supplemented with phytobiotics, HAL and AGPs.

Treatment							
Microorganism	NC	PC	HAL	Po8	Pb4	SEM	*p*-Value
*E. coli*	6.32 ^a^	5.82 ^b^	5.87 ^b^	5.86 ^b^	5.71 ^b^	0.04	<0.0001
*Total anaerobic bacteria*	5.92 ^a^	5.70 ^ab^	5.90 ^a^	5.84 ^ab^	5.60 ^b^	0.04	0.032
*Staphylococcus aureus*	2.60 ^a^	1.21 ^c^	2.56 ^a^	1.80 ^b^	1.27 ^c^	0.09	<0.0001
*Pseudomonas aeruginosa*	4.80	4.70	4.78	4.75	4.65	0.04	0.75
*Lactobacillus* spp.	6.23 ^b^	6.40 ^b^	6.41 ^b^	6.89 ^a^	6.98 ^a^	0.05	<0.0001
*Clostridium* spp.	5.20 ^a^	4.88 ^b^	5.18 ^a^	5.12 ^a^	4.90 ^b^	0.04	0.003
*Salmonella*	3.01 ^a^	2.27 ^c^	2.65 ^b^	2.70 ^b^	2.23 ^c^	0.05	<0.0001
*Cecum* pH	6.68	5.99	6.23	6.07	5.87	0.07	0.001

Note: ^a–c^ means with different superscripts in the same row differ significantly (*p* < 0.05). NC: (basal diet; negative control); PC: (BD + 0.2 g/kg tetracycline; positive control); HAL:(BD + 0.03 g/kg halquinol); Po8: (BD + POLM 8 g/kg); Pb4 (BD + PBLM 4 g/kg). SEM: standard error of mean.
